# Human Parvovirus 4 in Nasal and Fecal Specimens from Children, Ghana

**DOI:** 10.3201/eid1810.111373

**Published:** 2012-10

**Authors:** Jan Felix Drexler, Ulrike Reber, Doreen Muth, Petra Herzog, Augustina Annan, Fabian Ebach, Nimarko Sarpong, Samuel Acquah, Julia Adlkofer, Yaw Adu-Sarkodie, Marcus Panning, Egbert Tannich, Jürgen May, Christian Drosten, Anna Maria Eis-Hübinger

**Affiliations:** University of Bonn Medical Centre, Bonn, Germany (J.F. Drexler, U. Reber, D. Muth, A. Annan, F. Ebach, C. Drosten, A.M. Eis-Hübinger);; Bernhard Nocht Institute for Tropical Medicine, Hamburg, Germany (P. Herzog, J. Adlkofer, E. Tannich, J. May);; Kumasi Centre for Collaborative Research in Tropical Medicine, Kumasi, Ghana (A. Annan, N. Sarpong, S. Acquah);; Kwame Nkrumah University of Science and Technology, Kumasi (Y. Adu-Sarkodie);; and Freiburg University Medical Center, Freiburg, Germany (M. Panning)

**Keywords:** human parvovirus 4, PARV4, viruses, human partetravirus, genotype 3, Africa, Ghana, children, nasal swabs, feces, transmission

## Abstract

Nonparenteral transmission might contribute to human parvovirus 4 (PARV4) infections in sub-Saharan Africa. PARV4 DNA was detected in 8 (0.83%) of 961 nasal samples and 5 (0.53%) of 943 fecal samples from 1,904 children in Ghana. Virus concentrations ≤6–7 log_10_ copies/mL suggest respiratory or fecal–oral modes of PARV4 transmission.

Human parvovirus 4 (PARV4; human partetravirus) is a single-stranded DNA virus discovered in 2005 (*1*). PARV4 has been detected in persons at risk for parenteral infections, suggesting blood-borne transmission (*2*,*3*) although other transmission routes have not been ruled out. Studies in northern Europe demonstrated a high prevalence of antibodies against PARV4 in injection drug users, persons co-infected with HIV and hepatitis C virus, and persons with hemophilia who were exposed to nonvirally inactivated clotting factors; however, antibodies were not detected in the general population (*4*,*5*).

In contrast, PARV4 seroprevalence was 25%–37% in adults in the Democratic Republic of Congo, Cameroon, and Burkina Faso who were not infected with HIV and hepatitis C virus (*6*). PARV4 DNA was detected in blood of 8.6% of children 15 or 24 months of age in Ghana (*7*). There was no history of exposure to multiple-use needles or blood transfusion in any of these children. These data suggested alternative modes of PARV4 transmission in countries in Africa. Nonparenteral modes of transmission of PARV4 have also been suggested in South Africa (*6*), Taiwan (*8*), India (*9*), China (*10*), and Thailand (*11*).

PARV4 has been classified into 3 genotypes. Genotypes 1 and 2 are found in North America, Europe, and Asia (*1*–*3*,*9*–*11*), and genotype 3 is found in in sub-Saharan Africa (*7*,*12*). To investigate whether PARV4 is found in the respiratory or intestinal tract, we analyzed previously collected specimens from 1,904 children in Ghana.

## The Study

Ethical approval for this study was provided by the Committee on Human Research Publication and Ethics, Kwame Nkrumah University of Science and Technology, Kumasi, Ghana. Informed consent was obtained from parents or guardians of all children.

A total of 1,904 anonymous nasal and fecal specimens were obtained during a study on molecular diagnostics for respiratory and enteric tract infections in symptomatic children <15 years of age at the Presbyterian Hospital in Agogo, Ghana. Nasal swab specimens were obtained from children with upper or lower respiratory tract symptoms. Fecal samples were obtained from 504 children with gastrointestinal symptoms (53.4% of sampled children; 294 [58.3%] of symptomatic children with vomiting, 190 [37.7%] with diarrhea, and 144 [28.6%] with acute malnutrition; 9 [1.8%] with incomplete clinical data) and 439 (46.6%) children without gastrointestinal symptoms.

A total of 961 nasal swabs were obtained during February–November 2008 from 520 boys and 441 girls (median age 19 months, range 0–162 months, interquartile range 8–38 months). Nasal swabs were placed in 1.5 mL of RNAlater (QIAGEN, Hilden, Germany). A total of 943 fecal samples were obtained during May–October 2009 from 500 boys and 443 girls (median age 36 months, range 0–165 months, interquartile range 17–78 months). Fecal samples were prepared as 10% suspensions in phosphate-buffered saline. No paired nasal and fecal specimens were available from individual patients.

Viral DNA was purified from 140 µL of nasal swab suspension or 200 µL of fecal suspension by using QIAamp Viral RNA and DNA Stool Mini Kits (QIAGEN), respectively. Two real-time PCRs were performed. One primer/probe set was designed to detect PARV4 genotypes 1 or 2 viruses (*13*), and a second primer set was designed to detect PARV4 genotype 3 viruses (*7*). The sensitivity of both protocols was 1–2 genome copies/reaction. Absolute quantification of PARV4 genome copy numbers relied on photometrically quantified genotype 3 plasmid standards, as described (*7*).

To exclude bias from DNA purification methods, PARV4-negative nasal and fecal specimens were spiked with quantified plasmid standards. Subsequent quantification was equivalent between techniques and specimens, and differences between specimen types in several experiments were <0.5 log_10_ copies/mL. Standard procedures were used to prevent PCR contamination. Determination of PARV4 genotypes was conducted by nucleotide sequencing of several genomic target regions (Table).

**Table:** Nucleotide sequence divergence of parvovirus 4 strains from nasal swab and fecal samples from children, Ghana, from genotype 1, 2, and 3 prototype strains*

Specimen type and no.	Nucleotide position according to GenBank accession no. EU874248	Nucleotide sequence divergence from parvovirus 4 reference strains, %		
		Genotype 1 (GenBank AY622943)	Genotype 2 BR10627–5 (GenBank DQ873390)	Genotype 3 NG-OR (GenBank EU874248)
Nasal swab				
N1	1700–4660	6.56	7.39	0.92
N2	299–4660	7.51	8.07	0.88
N3	50–4660	7.37	8.38†	0.83
N4	1962–2056‡	9.16	6.73	2.14
N4	2117–3413	4.97	5.31	0.93
N5	1962–2056	9.16	6.73	2.14
N5	2117–4183	5.50	6.34	0.98
N6	299–4660	7.51	8.10	0.90
N7	1962–2056	9.16	6.73	2.14
N7	2431–2914	6.24	7.01	1.25
N7	3068–3246	4.61	5.19	1.12
N8	624–3246	7.36	7.84	0.84
Feces				
F1	1700–4183	6.20	6.82	0.89
F2	1700–4460	6.56	7.39	0.92
F3	1700–3716	6.08	6.52	0.85
F4	1700–4183	6.02	6.78	0.89
F5	1700–4183	6.93	6.73	1.04

*Pairwise nucleotide divergence was calculated by using the DNA distance matrix in BioEdit (www.mbio.ncsu.edu/BioEdit/bioedit.html). †Because the homologs of the first 92 nt of strain N3 are not given in the prototype strain BR10627–5, calculation of divergence started at N3 nt position 93. ‡Nucleotide sequence of the PCR product (primer sequences trimmed) was amplified by using screening PCR designed for detection of PARV4 genotype 3 as described (*7*).

**Figure 1:**
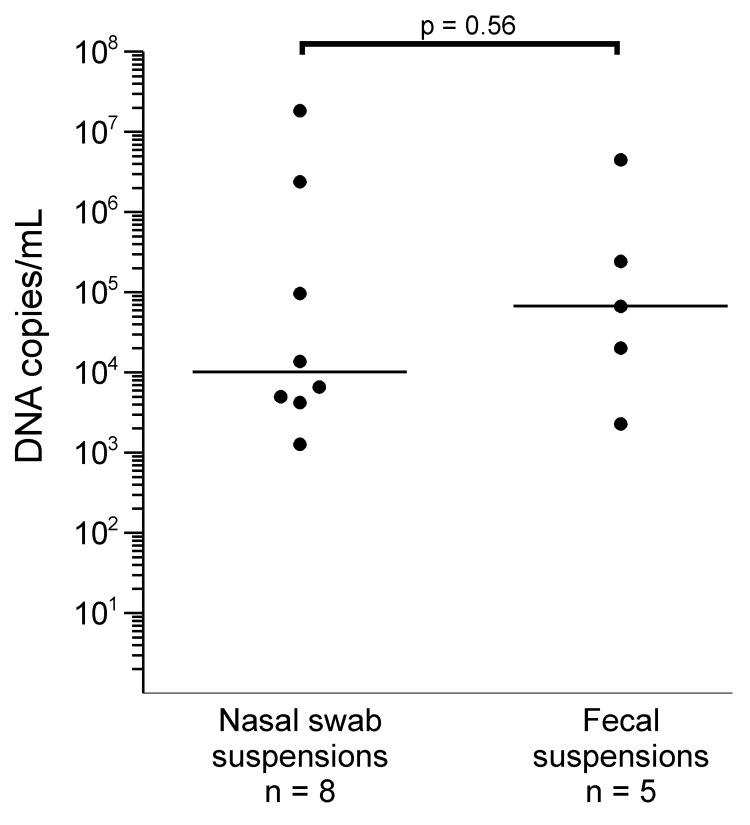
Parvovirus 4 DNA loads in virus-positive nasal and fecal specimens from children, Ghana. Virus concentrations are given on a log scale on the y-axis. Each dot represents 1 specimen. Horizontal lines represent median values for each sample type. For calculation of statistical significance of the difference in viral quantities between sample types, the Mann-Whitney U test was used. Virus quantities in nasal swabs and feces are given for sample suspensions (nasal swabs in 1.5 mL of stabilizing reagent and feces in a 10% suspension in phosphate-buffered saline).

Eight (0.83%) of 961 nasal swabs and 5 (0.53%) of 943 fecal samples tested were positive for PARV4 DNA. Virus concentrations ranged from 1.3 × 10^3^ to 1.8 × 10^7^ copies/mL (median 1.0 × 10^4^ copies/mL) in nasal swab suspensions and from 2.3 × 10^3^ to 4.6 × 10^6^ copies/mL (median 6.8 × 10^4^ copies/mL) in fecal suspensions (Figure 1). The difference in virus concentrations between the 2 groups was not significant (p = 0.056, by Mann-Whitney U test).

**Figure 2:**
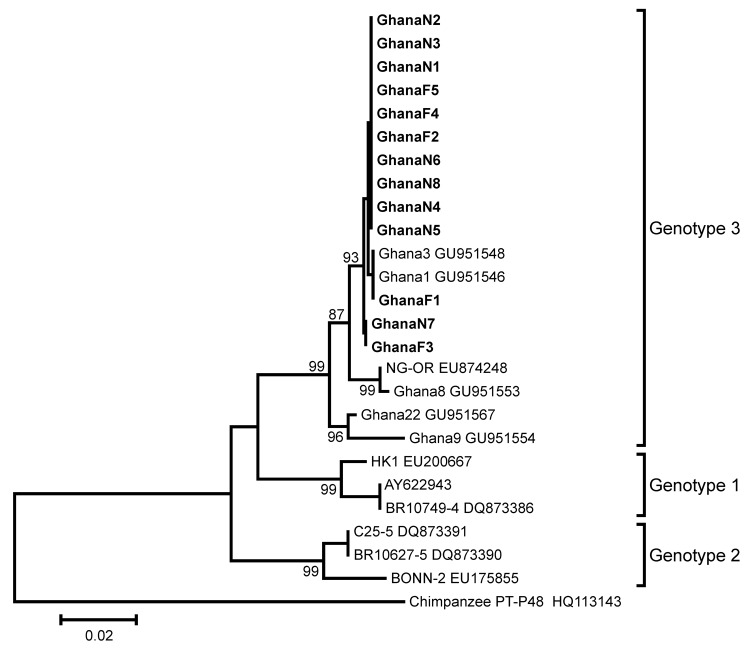
Phylogenetic analysis of a 483-nt fragment of the parvovirus 4 (PARV4) capsid-encoding open reading frame (ORF) 2 for PARV4 strains identified in children, Ghana. Neighbor-joining phylogeny was conducted in MEGA5.05 (www.megasoftware.net) by using a gap-free ORF2 fragment corresponding to positions 2,432–2,914 in the PARV4 genotype 3 prototype strain NG-OR (GenBank accession no. EU874248) with a nucleotide percentage distance substitution model and 1,000 bootstrap replicates. Scale bar indicates percentage uncorrected nucleotide distance. Previously published PARV4 sequences are given with strain names (if available) and GenBank accession numbers. Viruses newly identified are in **boldface**. The source of PARV4 strains identified in the study is indicated by capital letters (N, nasal specimen; F, fecal specimen). PARV4 genotypes are given to the right of taxa. A chimpanzee partetravirus was used as the outgroup.

Nucleotide sequencing of amplicons generated by screening PCRs and sequencing of additional genomic regions classified all viruses as PARV4 genotype 3 (Table) (GenBank accession numbers JN183920–JN183932). This result was confirmed by phylogenetic analysis of a 483-nt fragment of the capsid-encoding open reading frame 2 (Figure 2).

Ages of the 8 children with PARV4-positive nasal swab specimens ranged from 9 to 58 months (median 32 months). Ages of the 5 children with PARV4-positive fecal samples were 1, 36, 43, 57, and 124 months. Nasal swab specimens with the highest viral loads were from a 9-month-old boy and a 29-month-old girl. Fecal samples with the highest viral loads were from 2 boys 43 and 57 months of age.

## Conclusions

We found PARV4 in 0.8% of nasal swab specimens and 0.5% of fecal specimens from 2 groups of children in Ghana symptomatic for respiratory illness and with or without diarrheal illness, respectively. Our results provide evidence to suggest that the higher prevalence of PARV4 reported among adults in countries in western Africa (*6*) might be caused by transmission by the respiratory or fecal–oral route.

However, demonstration of PARV4 in the respiratory tract and feces does not identify a transmission route. PARV4 in the respiratory tract could be caused by high viremia, which was recently reported in a child in India with a genotype 2 infection (*9*) and in 2 patients with hemophilia in the United Kingdom, 1 with a genotype 1 infection and 1 with a genotype 2 infection (*14*).

It is unclear to what extent the putative nonparenteral transmission routes of PARV4 genotype 3 in western Africa apply to other areas. Markedly lower PARV4 antibody prevalences observed in Europe (*4*,*5*) argue against PARV4 spread by nonparenteral routes, e.g., from infected injection drug users to the general population. Likewise, the higher prevalence of PARV4 antibodies in HIV-infected blood donors in South Africa compared with uninfected donors (*6*) appears incompatible with PARV4 transmission primarily by the respiratory route. Therefore, our results do not contradict those of a study conducted in Scotland, which showed no PARV4 in respiratory specimens (*15*).

Because of the small number of children with PARV4 DNA in nasal or fecal specimens, correlation of infection with age groups was not possible. A limitation of our study was the lack of blood specimens from children with current respiratory or fecal PARV4 shedding, and serologic studies are needed to evaluate susceptibility of different age groups to PARV4 infection. Furthermore, detection of PARV4 in patients with respiratory disease does not indicate that PARV4 was the cause of the disease. In 5 of 8 PARV4-positive nasal swabs, typical respiratory viruses (parainfluenza virus, influenza A virus, rhinovirus) were also detected and the pattern of symptoms in PARV4-positive children did not differ from symptoms in PARV4-negative children. Similarly, 3 of 5 children with PARV4-positive feces did not have gastrointestinal symptoms at the time of fecal sampling. One child had vomiting and another child had vomiting and diarrhea. Moreover, in 3 of these 5 children, in addition to PARV4, *Giardia lamblia*, a potential cause of diarrhea, was also detected.

Although data for exposure and risk factors and paired samples were not available, suggested transmission routes might explain the high infection rates in western Africa. Further studies are needed to assess the effect of PARV4 excretion on virus epidemiology and the chronology of PARV4 infection.
